# Diagnosing cancer in patients with ‘non-alarm’ symptoms: Learning from diagnostic care innovations in Denmark

**DOI:** 10.1016/j.canep.2018.03.011

**Published:** 2018-06

**Authors:** Alice S. Forster, Cristina Renzi, Georgios Lyratzopoulos

**Affiliations:** Research Department of Behavioural Science and Health, UCL, Gower Street, London, WC1E 6BT, United Kingdom

**Keywords:** Cancer patient pathway, Non-specific symptoms, Diagnostic yield

Efforts to improve cancer outcomes have led to the introduction of policies to enable ‘fast-track’ referrals from primary to secondary care for patients with possible cancer. Although these schemes, also known as ‘two-week-wait’ referrals, have been successful in shortening diagnostic intervals [1,2], their usefulness is limited to patients who present with ‘alarm’ symptoms of relatively high predictive value for neoplastic disease [1,3]. In contrast, achieving a prompt diagnostic resolution in the approximate half of all cancer patients who initially present with non-specific and lower risk symptoms remains a challenge [1,2]. To address this problem, hospital-based multi-disciplinary diagnostic services have been recently introduced in Denmark and England for patients with non-specific symptoms [4,5]. Two Danish studies published recently in this Journal add substantially to relevant emerging evidence [6,7].

An important characteristic of a diagnostic service is its diagnostic yield (also known as ‘risk level’ or ‘conversion rate’) for detection of cancer. In principle, unselected patients with non-specific symptoms would be expected to have diagnostic yields for cancer that are lower to that observed for patients with symptoms of relatively high predictive value (in England, currently 8% of patients investigated for ‘alarm’ symptoms are found to have cancer, https://fingertips.phe.org.uk). In contrast, both recent studies in this Journal and earlier evaluations of Danish diagnostic centres for patients with non-specific (non-alarm) symptoms report diagnostic yield estimates for cancer ranging from 11% to 21%, clearly exceeding the values observed in English patients with alarm symptoms [[6], [7], [8], [9], [10], [11]] (Table 1). Understanding the likely underlying mechanisms by which these relatively high diagnostic yields are achieved is critical to enable the most useful translation of the pioneering Danish experience to other healthcare system settings.

**Table 1:** Characteristics of studies evaluating non-specific symptom pathways.

**Lead author, year**	**Criteria for entry to pathway**	**Triage in primary care**	**Triage in secondary care**	**Setting**	**N**	**Time period**	**Diagnostic yield for cancer**	**Diagnostic yield for other diagnoses**
Bislev et al. [8]	“Patients with serious non-specific symptoms and signs of cancer”	GP performs blood & urine tests, CT of thorax, abdomen & pelvis “prior to further evaluation and diagnostics at the hospital”	First, doctor, nurse and patient go through medical history/symptoms. Then physical examination and previously performed tests reviewed. Then individual plan for patient prepared	Aarhus hospital, Denmark	323	2011–2013	18%	33%
Ingeman et al. [9]	“Patients with serious non-specific symptoms” “if cancer is suspected although no alarm symptoms”	Depending on hospital location: GP performs blood & urine tests, plus abdominal ultrasound & chest X-ray; or CT of chest, abdomen & pelvis. Referral to diagnostic unit based on findings of these tests	Diagnostic unit conducts further investigations (e.g. blood tests, diagnostic imaging, endoscopies and biopsies) on basis of symptoms/clinical findings. Easy access to range of medical specialists	Aarhus & Silkeborg hospitals, Denmark	1,732	2012–2013	16%	Not reported
Moseholm et al. [10]	“Patients with serious non-specific symptoms and signs of cancer”	Not described	Not described	Four hospitals in the Capital Region of Denmark	1,127	2013–2014	20%	Not reported
Jørgensen et al. [11]	“Patients with non-organ-specific symptoms and signs of cancer, who were healthy enough for an outpatient course”	GP or other hospital department refers patients	Blood test & chest x-ray performed prior to visit for diagnostic unit. “On basis of information available at referral the physician” determines tests required before appointment. Further investigations planned at first visit	North Zealand hospital, Denmark	825	2013–2014	17%	Not reported
Moseholm and Lindhardt [6]	“Patients suspected of having cancer due to serious non-specific symptoms”	GP performs diagnostic imaging & blood & urine tests & referred to diagnostic unit if relevant (protocol varies across Denmark)	Facility for medical investigation and easy access to specialists	All referrals to NSSC-CPP, Denmark	23,934	2012–2015	11%	34%
Næser et al. [7]	Patients with non-specific symptoms	GP performs blood test & combined thoracic x-ray & ultrasound of abdomen. CT of chest, abdomen & pelvis if radiologist deems relevant. “GP initiates the diagnostic workup on basis of the” results. “If the triage function yields no obvious explanation for the patient’s symptoms”, GP refers to diagnostic centre	Centre run by internal medicine specialists. Individual diagnostic programmes based on medical history and results of previous investigations. All medical specialties represented at the centre. Centre has preferential arrangements with specialists to speed up investigations. Concurrent work up in different medical specialities may occur	Silkeborg hospital, Denmark	938	2012–2014	13%	22%
Nicholson et al. [12]	Patients aged ≥ 40, for whom there is no alternative suitable urgent referral pathway and presenting with one of six pre-specified non-specific symptoms/clinical findings (including ‘gut feeling’)	GPs access triage tests for these patients (blood tests, faecal immunochemical testing and low-dose computerised tomography). Based on the findings of these tests, patients are referred via an urgent referral pathway for cancer, or for further investigations	If cause of the symptoms remains uncertain, patient referred to a multidisciplinary diagnostic centre, with the investigations used here determined on an individual basis by the responsible clinician	Oxfordshire, UK	Ongoing	2017–current	Ongoing	Ongoing

Danish studies describe the presence of substantial pre-referral investigative activity (triaging) which involves the use of several primary care tests before a decision to refer patients with non-specific symptoms to multi-disciplinary diagnostic centres. Relevant triage investigations include blood and urine tests and diagnostic imaging, including CT scans (see Table 1 – column entitled ‘Triage in primary care’) [[6], [7], [8], [9],^11^]. This triaging process results in the selection of a patient group at appreciable risk of cancer - or other serious illness.

Beyond the critical role of primary care triage, some patients referred to multi-disciplinary diagnostic centres are reported as having symptoms of relatively high predictive value. This is in spite of the intended primary use of these services for investigation of patients with non-specific symptoms. As reported recently in this Journal, Næser et al. indicate that although the great majority of all referred patients (85%) had non-specific symptoms of low predictive value (such as weight loss, fatigue, or malaise), nearly 6 out of 10 referred patients had one or more ‘focal’ symptoms including certain ‘alarm’ symptoms, such as change in bowel habit (present in 18%), lump (6%), blood in stool (4%) and dysphagia (4%) [7].

Therefore, the diagnostic yields reported for Danish multi-disciplinary diagnostic centres are unlikely to simply reflect the diagnostic reasoning skills or the clinical intuition of the referring primary care physicians [12,13]. Rather, patients referred into the Danish non-specific symptoms pathway seem to represent a population at relatively high risk of cancer, selected through smart, primary care triaging.

Further elucidation of ‘risk enrichment’ mechanisms can enable the most useful translation of the pioneering Danish experience to other country contexts. It is particularly important to establish how the likelihood ratios of combinations of signs, symptoms and abnormal test results, could inform referral and investigation algorithms, to optimise cost-effectiveness and volume of referrals [14]. It is hoped that the ongoing evaluations of the recently developed English multi-disciplinary diagnostic centres will help to generate valuable relevant evidence in the near future [12] (Table 1).

Another key question about diagnostic services for patients with non-specific symptoms is their likely contribution in promptly detecting consequential illness other than cancer. Both recently published studies from Denmark indicate that between a fifth and a third of all patients referred to multi-disciplinary diagnostic centres were diagnosed with clinically significant non-neoplastic disease [6,7]. Moseholm and Lindhardt report that among referred patients, 9% were found to have cardiovascular disease, 7% gastrointestinal disease, and 5% musculoskeletal and connective tissue disorders [6]. Although further establishing the diagnostic yield for non-neoplastic diagnoses, and their spectrum, remains important, patients are likely to value a prompt diagnostic resolution independently of whether a formal diagnosis is reached or excluded as a source of their symptoms.

The speed by which diagnostic resolution can be achieved in patients with non-specific symptoms is of great interest to both patients and planners of healthcare services. Moseholm and Lindhardt [6] report that diagnostic resolution in the evaluated Danish diagnostic centres was achieved after an average of four outpatient appointments during a seven-day period, noting that in a substantial minority of patients the diagnostic process was longer. These findings provide for a ‘reality check’ about the concept of ‘one-stop / same-day’ evaluations, which seem unlikely for most referred patients. However, reported diagnostic intervals are substantially shorter than those likely to have been experienced in the absence of a scheme for the referral of patients with non-specific symptoms.

Multidisciplinary diagnostic services represent complex healthcare interventions [15]. Eligible patients have heterogeneous symptoms and disease states, and the use of various investigations at multiple time points from presentation to diagnosis creates a very high number of combinations of test sequences. Evaluating these complex healthcare interventions using controlled study designs would be ideal. Studies should provide evidence about selection criteria regarding presenting symptoms and triaging tests, and the diagnostic strategies (both within primary care and once the patient has been received at the diagnostic centre) associated with optimal diagnostic yield, patient safety and cost-effectiveness. It is important to collect follow-up data on treatment and prognosis (survival), and explore the clinical and psychological impact of tests on patients who are referred and investigated but found to have no important illness. The risks of over-diagnosing asymptomatic disease unrelated to the ‘trigger’ symptomatic presentations which would not have presented clinically during the patient’s lifetime should also be quantified. Cost-effectiveness studies are needed to balance potential cost savings from fewer appointments and potential earlier diagnosis against the fact that many individuals will receive expensive diagnostic healthcare without obvious clinical benefit and at risk of potential harm.

In conclusion, the studies by Moseholm and Lindhardt [6] and Næser et al. [7] recently reported in this Journal demonstrate relatively high yields regarding the diagnosis of cancer and other important illness following referral to hospital-based diagnostic services for non-specific symptoms. Taken together with other evidence from Denmark, these studies highlight useful mechanisms by which the effectiveness of such services can be optimised, critically involving an active role of triage in primary care. Future research, ideally using controlled designs, should aim to more fully characterise the presenting symptoms of referred patients and refine the most effective diagnostic test cascades, together with optimal triaging protocols in both primary and secondary care. Controlled studies evaluating these multi-faceted complex interventions, including process evaluations exploring the mechanisms by which they work, can help to elucidate their potential effectiveness in different clinical scenarios, and help to improve the diagnostic experience and outcomes for the half of cancer patients who initially present to primary care without alarm symptoms.

## Authorship contribution

All authors had a substantial contribution to the concepts outlined in this commentary; in drafting the article and have approved the final version to be published.

## Conflicts of interest statement

None.
